# The Proofs of Infanticide Considered

**Published:** 1836-10-01

**Authors:** 


### The Proofs of Infanticide considered. Including Dr. Hunter's Tract on Child-Murder, with Illustrative Notes, and a Summary of the present State of Medico-Legal Knowledge on that Subject. By *William Cummin*, M.D. &c. &c. Lecturer on Forensic Medicine. Duodecimo, pp. 95. Longman's, August, 1836.

There is no doubt that infanticide is one of the most puzzling problems in medical jurisprudence. Dr. William Hunter, in his eloquent attempt to cut the Gordian Knot, has really mystified the subject, and rendered the solution of the question more difficult. If, indeed, the whole female world had clubbed to fee an advocate for pleading their cause, in case of frail slips, and certain disagreeable consequences thence occasionally resulting, they could not have employed a more powerful or successful one than Dr. William Hunter. No one can peruse his tract, without seeing the special pleader in almost every page. We do not blame him. He was on the side of humanity and mercy, though his arguments have often defeated rigid justice. Be that as it may, his Essay is the Koran of the Bench and the Bar. The Judge quotes it with implicit faith, and the counsellor cross-examines the witnesses on the difficulties which it portrays. It requires some brass, and it involves some danger, to openly and confidently oppose the sentiments of a Hunter on the subject of infanticide. To do so, a witness must have all his wits about him. He must be familiar with Dr. Hunter's tract itself, and he must be conversant with the Doctor's weak points and errors, as exposed by modern investigations and experiments. Both these objects will be obtained by a study of Dr. Cummin's little volume. In the first place, he has republished the text of Dr. Hunter's Essay—in the second place, he has appended notes to it in order to estimate the value of Dr. H's objections to all tests (for this is the tendency of his Essay) in ordinary cases of child-murder. In the third place, he has given, by way of summary, a comprehensive view of all the points specially connected with an investigation of the proofs and tests of infanticide. But it is not sufficient that such things are done, unless they are done well. We think they are done well—and that this little vade mecum contains the quintescence of all that has been written on the subject of child-murder. We should be very sorry to go into a witness-box without first conning over every page of this little manual.

To offer concentration of what is already compressed to the smallest possible space, would be ridiculous, and a specimen is very inadequate to convey an idea of the performance. Yet we have no alternative, and the following extract relative to the hydrostatic test, is important in itself, and one that may be more properly insulated from surrounding matters than most others.

THE HYDROSTATIC TEST.

" In order to apply this test properly, we ought to have a vessel (such as a glass jar) of sufficient capacity to contain both the lungs and heart, and to suffer them freely to float or sink, according to circumstances. Pure river or rain water should be used, and its temperature should neither be too low nor too high —say 60° Fahr. The vessel being nearly full, let the lungs and heart and the thymus be removed from the chest (taking care previously to tie the large blood-vessels), and let the whole be placed in the water together.

We should then observe whether the parts, thus collectively, swim or sink. If the latter, whether they reach the bottom quickly or slowly. The lungs may then be separated from the heart, and tried one after the other, as to their tendency to sink or remain buoyant. They may next be cut in pieces, and examined in detail as to their gravitating properties; and ultimately the fragments may be pressed so as to expel the air as completely as possible.

Now the chief and most obvious inferences derived from the hydrostatic test, are, 1st, that if the lungs swim, the infant breathed, or was born alive; 2dly, that if they sink, the infant did not breathe—it was still-born.

1. *Swimming of the Lungs.*

If the lungs, wholly or in part, swim, the inference is, that respiration to some extent, more or less, has taken place; but there are certain doubts or objections which may be raised, and are to be removed before our absolute decision is pronounced.

*Objections.*

(*a.*) *Putrefaction* may cause the lungs to float. This is true, in certain circumstances: when the lungs are in that state of putrefaction in which gases are generated, and cannot immediately escape, they are buoyant, and swim on water. But putrid lungs do not always float; their putridity may have attained such a degree as to keep them at the bottom, even though they had once contained air in their air-cells. In the latter case, by the way, the hydrostatic test is of no use; and neither will any other test avail; for the infant's body must, in such a state of things, be one mass of corruption.

But in ordinary cases submitted to the medico-legal examiner—say within a few hours or days after birth—there can be no room for even supposing the presence of putrefaction. In fact, the lungs are known to be among the last, if not the very last, parts of the body which become putrid; and it has often happened, that when the body of an infant has been found in a state of putridity, the lungs have still been fresh, and fit for the application of the proper tests.

When, however, there is reason to suspect that the lungs float owing to this cause, let them be examined; and if putrid gases are really developed, rendering them specifically lighter than water, those gases will be seen, in large blister-like vesicles, on the surface and between the lobules of the lungs; whence they may be readily expelled by pressure under water. For greater certainty, the lungs may be cut in pieces, and each piece pressed separately; such pressure will not destroy the buoyancy of the parts, if they have ever received atmospheric air into their cells; whereas they sink at once if they had floated through mere putridity.

(*b.*) *An emphysematous condition* may cause the floating of the lungs. It sometimes happens that infants suffer violence in the birth; the labour, perhaps, being tedious, and the mother malformed. The sides of the chest may be so pressed against the substance of the lungs as to do those organs injury: they become inflamed and puffy, containing air in large vesicles on their surface; and this is what some authors call emphysema. When such a state does exist, it may be recognized by the experienced eye, and by the superficial air expelled, as in the case of the putrid gases. No serious obstacle consequently can arise from this cause to the application of the hydrostatic test.

(*c.*) *Air may have been artificially introduced.* In the notes to Dr. Hunter's tract (p. 35, *ante*), this objection has been pretty fully considered. The *static* test may here be brought to our aid. Air blown into the lungs of a still-born child never produces all the changes which follow natural respiration. The moment the atmospheric air is admitted into the lungs of a living child, those organs are expanded, and their vessels filled with blood from the heart. Their weight consequently is increased,—doubled, as we have seen in some instances: so that by compaing the weight of the lungs with the weight of the body, a presumption of much importance may be formed. Again: another peculiarity by which the effects of inflation may be distinguished from those of respiration, is, that strong and energetic pressure will cause to sink every particle of lungs artificially inflated, while no mere pressure, short of absolute breaking up and mashing of the parts, will cause those lungs wholly to sink with which an infant has naturally respired.*

The objection that artificial inflation might have been practised, it must be confessed, may sometimes present the medical jurist with more than ordinary difficulty. But the circumstances of the case ought to save him from vexatious

---

* For this method of testing artificial inflation (already noticed, p. 37, *ante*), we are indebted to Mr. Jennings, of Leamington. See *Trans. Prov. Associat.* Vol. II. p. 435.

and needless investigation. The truth is, that such a plea on behalf of an accused mother is extremely rare, not only because, in most cases, it has no foundation in truth, but because, to render it probable, evidence of many collateral facts ought to be forthcoming. The female who would endeavour to save her child by inflating its lungs, should have given other proofs, besides, of her maternal tenderness : she should not have concealed, at least from some intimate friend, the fact of her pregnancy ; her delivery should not have been secret ; she should have prepared for the birth—the living birth—of her infant ; there should be no marks of wilful violence on the body : in short, it is easy to judge from the history of any given case, whether the accused *wished* the life or death of the child, and therefore whether it is likely she *would*, (even allowing that, with sufficient strength and self-possession at such a moment, she *could*) inflate the infant's lungs.

Another plea, for *humanity's* sake, suggested by Dr. Hunter and others, is that inflation might have been practised by some malicious person, in order to trump up a charge of infanticide against the mother. Now there happens never to have been, as we trust there never will be, an instance of this kind of malice. Only consider all that would be required to make it in any degree effective. The diabolical perpetrator of such a deed should have some medical and medico-legal knowledge ; his interference, too, could hardly have been premeditated—at least it must be contingent on the infant being born dead. In this case, too, there should be no collusion, or semblance of guilt, about the mother ; no secresy, no feigning, nor dissembling : in fine, no violence on the body of the child ; or should there be marks of violence, they should be such as were probably inflicted *after* death, not before it, as the infant is presumed to have been still-born ; so that, on a little reflection, it must be seen how purely frivolous and gratuitous—how almost utterly impossible, it would be to substantiate a plea of this kind, were it ever set up in a trial for infanticide. But, as we have said, we are not aware that it ever has been.

(*d.*) *The infant may have breathed in the passage.* It is beyond a doubt that an infant *may* breathe before it is wholly expelled from the mother ; but if it be so vigorous as to commence respiration thus early, what may have caused its death before it was wholly born ? Such cases are universally admitted to be rare, and when they do occur, to be owing to tedious delivery, or some physical obstacle on the part of the child, or the mother, in the way of malformation. If any thing of this kind have caused the infant's death, it ought so to appear in evidence. Respiration in the passages may also take place, where the child is born by the feet, and the head is detained for some time ; or where the hand is introduced to accelerate a tedious labour. But neither of these cases is likely to occur in the practice of legal medicine, for they imply the presence of medical or other assistance ; at least the circumstances preclude the idea of concealment. The objection, therefore, of breathing before delivery, ought not to be admitted without some feasible ground." 64.

We recommend this little work in the strongest manner.

